# Rubber Plates

**Published:** 1893-12

**Authors:** T. F. Skeede

**Affiliations:** Seward, Neb.


ARTICLE III.

### RUBBER PLATES.

BY T. F. SKEEDE, SEWARD, NEB.

Horace Greeley lived in the city of New York, and wrote a book entitled "What I Know About Farming." I live in the country, and will try to tell our city brethren what I know about rubber plates.

Being confined to a country practice, my patients are largely from the farming community, and cannot afford a continuous gum plate or the price of bridge-work. If we try to talk "continuous gum" or "bridge-work" to them for ten dollars a tooth, they hold up both hands in holy horror. With all due regard for the great and shining inventions, such as implantation, cutting off sound teeth for abutments for bridge-work, continuous gum at $75 a plate, etc., I know of no invention that has been of such benefit to suffering humanity as vulcanized rubber as a base of artificial teeth.

Professor Haskell will tell you that rubber plates will cause absorption of the alveola. Mr. President, "great men will differ," consequently I differ with Professor Haskell. I have here a cast showing absorption of the alveola, after wearing a temporary plate for eight years.

I have also a cast showing absorption of the alveola after wearing no plate at all for fourteen years.

Some will tell you that rubber is poison, causing diseases of the gum, or so-called "rubber" sore-mouth. Now, Mr. President, I do not believe there ever was a case of "rubber" sore-mouth, and I challenge any one to bring me a case of "rubber" sore-mouth that I cannot cure with a rubber plate.

The worst case I ever saw was under a small platinum plate that was put in the mouth in England, and had been worn four years without being removed. I removed the plate and found several very odoriferous substances, among them four roots, two of them ulcerated; also the liveliest case of "rubber" sore-mouth that ever came under my notice.

A gentleman came to me one day with a very sore mouth. He had been wearing a small rubber plate, and his physician had told him that the mercury in the rubber had salivated. I cleaned his plate, which he was then wearing in his pocket, gave him a small bottle of listerin, and told him to put the plate in and wear it, cleaning it three times a day. He was all right in a week, has worn his rubber plate over three years, and has not been "salivated since. It is not the rubber that makes the mouth sore, but the filth that is allowed to collect on and around it. A plate that is properly constructcd is easily and quickly cleaned with a little water containing a few drops of ammonia.

The first thing in making a rubber plate is a perfect impression, which should be as perfect as it is possible to get it. This "good enough" will not do, for nothing is good enough which can be made better by a reasonable amount of time and perseverance, I use plaster, modeling compound, or a combination of both, whichever will best answer the purpose. No one thing is best in all cases. Then get a perfect articulation, for as much depends on the articulation as on the fit of the plate. After I get my articulation, I examine the mouth thoroughly for hard and soft places. I then scrape my impression on the median line from front to back, scraping deepest where the mouth is hardest, cutting away well in the front part of the palatine surface, as Professor Haskell would say "to relieve pressure," but in reality making an air-chamber, and relieving the pressure at the same time. After pouring my model and separating, I scrape the model where the mouth is soft, deepest where the mouth is softest. I then cut a groove across the palatine arch, near the back edge of the plate, and continue it entirely around the model, near where the upper edge of my plate will come, thus making an additional air-chamber of the whole plate. For my base plate I use common tea lead, using two or three thicknesses, as occasion requires, which, by the way, does not require more than half the thickness usually given to rubber plates, waxing where thickness is needed. After flasking and separating I remove all wax carefully, then usually cover both casts with very thin tin foil, but always cover the model. Next I soap well and pack, thus vulcanizing my rubber between metal, insuring a nearly finished plate when it comes from the vulcanizer. I never boil my rubber plates.

One of the greatest objections to rubber is that it shrinks in cooling, and creeps away from the teeth, leaving space for saliva, etc., to work in. If a plaster cast has been boiled in water at 320° for an hour, the plaster is so soft that it offers no resistance to the rubber. I use a tin blacking box cover, or something similar in the bottom of the vulcanizer, setting my flask on this, putting in but but one or two teaspoonsful of water. Nothing touches my flask but hot steam. The plaster comes out hard, and the rubber firm against the teeth. A plate vulcanized between metal will have a harder surface than can be put on it in any other way. If cleaned semi-occasionally, it will never make the mouth sore, unless there is a misfit; and when the alveola has absorbed so the plate no longer fits, it is time to give some fellow a job of making a new one.—*Items of Interest.*

